# Potential role of insulin receptor isoforms and IGF receptors in plaque instability of human and experimental atherosclerosis

**DOI:** 10.1186/s12933-018-0675-2

**Published:** 2018-02-20

**Authors:** Nuria Beneit, José Luis Martín-Ventura, Carlota Rubio-Longás, Óscar Escribano, Gema García-Gómez, Silvia Fernández, Giorgio Sesti, Marta Letizia Hribal, Jesús Egido, Almudena Gómez-Hernández, Manuel Benito

**Affiliations:** 1 Biochemistry and Molecular Biology II Department, School of Pharmacy, Complutense University of Madrid, Plaza Ramón y Cajal s/n, 28040 Madrid, Spain; 2 Health Research Institute of San Carlos Clinic Hospital (IdISSC), Madrid, Spain; 3 CIBER of Diabetes and Associated Metabolic Diseases (CIBERDEM), Madrid, Spain; 4 Vascular Research Lab, IIS-Fundación Jiménez Díaz-Autonoma University, Madrid, Spain; 5 CIBER of Cardiovascular Diseases (CIBERCV), Madrid, Spain; 6 Department of Medical and Surgical Sciences, University Magna Graecia of Catanzaro, Catanzaro, Italy

**Keywords:** Atherosclerosis, Insulin receptor isoforms, Insulin-like growth factor receptor, Vascular smooth muscle cells, Apoptosis

## Abstract

**Background:**

Clinical complications associated with atherosclerotic plaques arise from luminal obstruction due to plaque growth or destabilization leading to rupture. We previously demonstrated that overexpression of insulin receptor isoform A (IRA) and insulin-like growth factor-I receptor (IGF-IR) confers a proliferative and migratory advantage to vascular smooth muscle cells (VSMCs) promoting plaque growth in early stages of atherosclerosis. However, the role of insulin receptor (IR) isoforms, IGF-IR or insulin-like growth factor-II receptor (IGF-IIR) in VSMCs apoptosis during advanced atherosclerosis remains unclear.

**Methods:**

We evaluated IR isoforms expression in human carotid atherosclerotic plaques by consecutive immunoprecipitations of insulin receptor isoform B (IRB) and IRA. Western blot analysis was performed to measure IGF-IR, IGF-IIR, and α-smooth muscle actin (α-SMA) expression in human plaques. The expression of those proteins, as well as the presence of apoptotic cells, was analyzed by immunohistochemistry in experimental atherosclerosis using BATIRKO; ApoE^−/−^ mice, a model showing more aggravated vascular damage than ApoE^−/−^ mice. Finally, apoptosis of VSMCs bearing IR (IRLoxP^+/+^ VSMCs), or not (IR^−/−^ VSMCs), expressing IRA (IRA VSMCs) or expressing IRB (IRB VSMCs), was assessed by Western blot against cleaved caspase 3.

**Results:**

We observed a significant decrease of IRA/IRB ratio in human complicated plaques as compared to non-complicated regions. Moreover, complicated plaques showed a reduced IGF-IR expression, an increased IGF-IIR expression, and lower levels of α-SMA indicating a loss of VSMCs. In experimental atherosclerosis, we found a significant decrease of IRA with an increased IRB expression in aorta from 24-week-old BATIRKO; ApoE^−/−^ mice. Furthermore, atherosclerotic plaques from BATIRKO; ApoE^−/−^ mice had less VSMCs content and higher number of apoptotic cells. In vitro experiments showed that IGF-IR inhibition by picropodophyllin induced apoptosis in VSMCs. Apoptosis induced by thapsigargin was lower in IR^−/−^ VSMCs expressing higher IGF-IR levels as compared to IRLoxP^+/+^ VSMCs. Finally, IRB VSMCs are more prone to thapsigargin-induced apoptosis than IRA or IRLoxP^+/+^ VSMCs.

**Conclusions:**

In advanced human atherosclerosis, a reduction of IRA/IRB ratio, decreased IGF-IR expression, or increased IGF-IIR may contribute to VSMCs apoptosis, promoting plaque instability and increasing the risk of plaque rupture and its clinical consequences.

**Electronic supplementary material:**

The online version of this article (10.1186/s12933-018-0675-2) contains supplementary material, which is available to authorized users.

## Background

Atherosclerosis is a chronic disease affecting large arteries that involves the formation of plaques containing vascular and inflammatory cells, lipids and extracellular matrix [1]. Its clinical complications arise from luminal obstruction due to plaque growth leading to vessel stenosis, and/or formation of unstable plaques that acutely rupture leading to an occlusive thrombus formation [2]. Vascular smooth muscle cells (VSMCs) play a main role in this process as they contribute to plaque growth in early stages, but favor plaque stability in advanced stages of atherogenesis [3].

The insulin and insulin-like growth factors (IGFs) signaling is mediated by hormone interaction with the insulin receptor (IR) and the IGF-I receptor (IGF-IR), which are members of subclass II tyrosine kinase receptor super-family [4, 5]. In mammals, alternative splicing of the IR gene gives rise to two isoforms: IRA and IRB [6]. Indeed, IRB has an additional 12-amino acid sequence encoded by the exon 11. Although both isoforms have similar affinity for insulin, IRA exhibits a higher affinity for IGFs, especially for IGF-II [7]. Thus, IRB is preferentially associated with metabolic and differentiating signals, whereas IRA mainly favors cell growth, proliferation and survival [8]. In addition to IR and IGF-IR, IGF-II binds IGF-II receptor (IGF-IIR) with high affinity. IGF-IIR is a type I transmembrane glycoprotein that also have high affinity for mannose-6-phosphate, and can therefore bind lysosomal enzymes and other growth factors and cytokines [9]. It plays a well-documented role in the intracellular transport of lysosomal enzymes and in clearance of IGF-II from the circulation. However, although IGF-IIR contains neither tyrosine kinase activity nor an autophosphorylation site, it does link to G-proteins providing a mechanism for signal transduction that may be involved in cell behavior regulation [10, 11].

In early atherosclerotic lesions, IGFs contribute to plaque growth by promoting VSMCs proliferation and migration [12]. In this regard, we previously demonstrated that overexpression of IGF-IR or IRA isoform during early atherosclerosis confers a proliferative and migratory advantage to VSMCs favoring atherosclerotic progression [13, 14]. In advanced stages, the imbalance between cell death and survival may substantially affect the cellularity and integrity of atherosclerotic lesions contributing to plaque instability. Unstable plaques that are prone to rupture have a thin fibrous cap with a decreased number of VSMCs and a dense infiltration of inflammatory cells [15, 16], as well as an increased apoptosis of VSMCs and macrophages [17]. IGF-I, through IGF-IR, has been reported to prevent atherosclerotic plaque instability by its mitogenic and antiapoptotic effects on VSMCs [18–20]. However, the role of IR isoforms or IGF-IIR in VSMCs apoptosis and thereby in plaque instability remains unknown. In the present study, we analyzed the expression of IR isoforms, IGF-IR and IGF-IIR, as well as VSMCs content in human carotid atherosclerotic plaques and in experimental models of atherosclerosis. Finally, we assessed the contribution of IR isoforms and IGF-IR to the apoptosis of murine aortic VSMCs lines.

## Methods

### Patients

Ten atherosclerotic plaques from patients undergoing carotid endarterectomy at IIS-Fundación Jiménez Díaz (Table 1) were dissected separating the stenosing complicated plaque (CP) from the non-complicated (NCP) fibrous adjacent area. The CP was defined as the lesion, usually localized at the origin of the internal carotid artery responsible for the surgery. The CP contained an important proportion of inflammatory cells (Stary stages V–VI), whereas the NCP adjacent areas were mainly composed of VSMCs and lipid deposits (Stary stage III). Masson’s trichrome stain of representative NCP and CP samples are shown. The study was approved by the hospital’s ethics committee (IIS-Fundación Jiménez Díaz) with the reference number (PI1442016) according to the institutional and the Good Clinical Practice guidelines, which was performed in accordance with the Declaration of Helsinki. All participants gave written informed consent.

**Table 1:** Clinical characteristics of patients bearing carotid atherosclerosis

Variable	Patients (n = 10)
Age, years	69 (57–78)
Gender (male/female), %	100/0
Diabetes mellitus, %	50
Hypertension, %	60
Dyslipidemia, %	50
Coronary artery disease, %	30
Current smoking, %	50

### Experimental models

Male mice were maintained on a 12-h light–dark cycle and 23 °C room temperature. All animals used are under C57BL/6 genetic background [21]. Generation of brown adipose tissue-specific insulin receptor knockout (BATIRKO); ApoE^−/−^ mice by crossing female ApoE^−/−^ mice with male BATIRKO mice was previously described [21]. Six week-old male Control mice, ApoE^−/−^ mice and BATIRKO; ApoE^−/−^ mice were fed on a standard diet (3% kcal from fat) for 62 weeks or a Western type diet (21% kcal from fat) for 18 weeks, and sacrificed at 24 weeks or 15 months of age, respectively. Anesthetized mice (Avertin, 250 mg/kg, ip) were saline-perfused. The aortic root was embedded in Tissue-Tek^®^ optimum cutting temperature (OCT) Compound and frozen for histological analysis, and the thoracic aorta was frozen for RNA extraction. All animal experimentation described in this manuscript was conducted according with accepted standards of human animal care, as approved by the institutional committee of Complutense University of Madrid (reference number: ES280790000086). All animal procedures have been performed according to the guidelines from Directive 2010/63/EU of the European Parliament and the National Institutes of Health (NIH) on the protection of animals used for scientific purposes.

### Cell cultures

Generation of immortalized IRLoxP^+/+^, IR^−/−^, IRA and IRB VSMCs lines was previously described [13]. Briefly, primary VSMCs were obtained from thoracic aorta arteries of 3 male 8-week-old IRLoxP^+/+^ mice. Anesthetized mice (Avertin, 250 mg/kg, ip) were saline perfused and thoracic aorta arteries were submitted to collagenase dispersion and primary culture. Then, primary culture of IRLoxP^+/+^ VSMCs were immortalized by transfection with pBabe retroviral vector encoding SV40 Large T antigen and selected with 1 μg/mL puromycin for 3 weeks. Immortalized IRLoxP^+/+^ VSMCs were infected with adenoviruses encoding Cre recombinase to obtain IR^−/−^ VSMCs. Finally, IR^−/−^ VSMCs were transfected with pBABE retroviral vector encoding the individual spliced isoforms of the human IR, IRA or IRB, and selected with 200 μg/mL hygromycin for 2 weeks to obtain IRA or IRB VSMCs respectively.

Cell lines were cultured to subconfluence (70–80%) with 10% foetal bovine serum (FBS)-DMEM for in vitro experiments. Cells were serum and glucose starved for 4–5 h and then treated with IGF-I (10 nmol/L, Merck Millipore), IGF-II (10 nmol/L, Merck Millipore), the IGF-IR inhibitor picropodophyllin (1 µmol/L, PPP, Merck Millipore), and/or thapsigargin (0.1–100 nmol/L, Sigma-Aldrich).

### Histological analysis

Paraffin-embedded human carotid atherosclerotic plaques were cross sectioned into 4 µm thick pieces, dewaxed, and rehydrated. Masson trichrome were performed following manufacturer’s instructions and as previously described [22].

Aortic roots from experimental models were OCT-embedded and sections of 7 μm interval were used for immunohistochemical studies. IR, IGF-IR, IGF-IIR and cleaved poly ADP ribose polymerase (PARP) were detected by immunoperoxidase with rabbit anti-IRβ (sc-711), anti-IGF-IRβ (sc-713) and anti-IGF-IIR (sc-25462) polyclonal antibodies and anti-Cleaved PARP (ab32064) monoclonal antibody. We also performed immunofluorescence against α-SMA using anti-actin, α-smooth muscle-Cy3™ mouse monoclonal antibody (C6198, Sigma-Aldrich) followed by 4′,6-diamidino-2-phenylindole (DAPI) staining to localize nuclei.

### Western blot analysis

Western blot analyses were performed on protein extracts from human plaques and from in vitro experiments as previously described [22]. The antibodies used were anti-IRβ, IGF-IRβ and IGF-IIR from Santa Cruz Biotechnology (Dallas, TX, USA); IRS-1 from Millipore (Billerica, MA, USA); p-IRS-1 (Ser307), p-AKT (Thr308), p-p42/44 MAPK (Thr202/Tyr204) and Cleaved Caspase-3 (Asp175) from Cell Signaling Technology (Danvers, MA, USA); anti-β-actin, α-SMA and α-tubulin from Sigma-Aldrich Corp. (St. Louis, MO, USA).

### Immunoprecipitation

An amount of 150 µg protein from human plaques was immunoprecipitated at 4 °C with IRB isoform antibody (provided by Dr. Sesti and Dr. Hribal). Supernatants were subsequently immunoprecipitated with IRβ antibody recognizing both IR isoforms. Thus, immune complexes from first (only IRB isoform) or second (only IRA isoform) immunoprecipitations were collected on protein A-agarose beads and submitted to SDS-PAGE. Finally, immunoblots were incubated with anti-IRB or anti-IRβ antibodies to analyze the expression of IRB and IRA, respectively. To study the association between IR isoforms and IGF-IR or insulin receptor substrate 1 (IRS-1), immunoblots were reincubated with anti-IGF-Iβ, IRS-1 or p-IRS (Ser307) antibodies.

### RNA extraction and real-time quantitative polymerase chain reaction

Total RNA was isolated from aorta of experimental models using TRIzol reagent (Invitrogen, Carlsbad, CA) and quantified by absorbance at 260 nm. One microgram of RNA was used to perform the reverse transcription with a High Capacity cDNA Archive kit (Applied Biosystems, Foster City, CA). Real-time quantitative PCR (qRT-PCR) was performed on an ABI Prism 7900 sequence detection PCR system (Applied Biosystems) according to the manufacturer’s protocol, using the ΔΔCt method as previously described [23]. Thus, the amount of target, normalized to endogenous gene and relative to the control, is given by real-time quantitative (RQ) = 2^−ΔΔCt^; ΔCt (cycle threshold) = Ct (target gene) − Ct (GAPDH); ΔΔCt = ΔCt for any sample − ΔCt for the control. Amplification of GAPDH was used in the same reaction of all samples as an internal control.

### Annexin V-FITC and propidium iodide assays

10^6^ cells were seeded in 60 mm-plates and the following day they were FBS deprived for 4 h and stimulated with IGF-I, IGF-II or PPP 1 h-previous to stimulation with thapsigargin for 18 h. After that, cells were counted and collected to perform apoptosis studies with Annexin V-FITC kit. Apoptotic cells are positively stained for Annexin V-FITC that binds to phosphatidylserine (PS) residues, but are negatively stained for propidium iodide (PI). Dead cells are positive for both, Annexin V-FITC and PI staining, whereas viable cells are negative for both Annexin V-FITC and PI. Finally, necrotic cells are positively stained for PI and negatively for Annexin V-FITC. For these experiments, a FACScalibur flow cytometer and Cell Quest Pro software (Becton–Dickinson) was used.

### Statistical analysis

All values are expressed as mean ± standard error of the mean (SEM). Differences between two groups were assessed using paired two-tailed *t*-tests for human samples and unpaired two-tailed *t*-tests for experimental models and in vitro experiments. Data involving more than two groups were analyzed using a one-way ANOVA followed by a Bonferroni test if differences were noted (GraphPad Prism 6.0). The null hypothesis was rejected when p < 0.05.

## Results

### Decrease of IRA/IRB ratio and IGF-IR, increase of IGF-IIR, and loss of VSMCs in complicated human atherosclerotic plaques

To assess the role of IR isoforms, IGF-IR and IGF-IIR in human advanced atherosclerosis, we used atherosclerotic plaques from patients undergoing carotid endarterectomy. Ten carotid endarterectomy samples were carefully dissected separating the stenosing complicated plaque (CP, Stary stages V–VI) from the non-complicated (NCP, Stary stages III) fibrous adjacent area. Histological analysis revealed that complicated plaques contained an intraplaque hemorrhage and/or a certain degree of calcification with a relatively important proportion of inflammatory cells. The adjacent non-complicated regions were composed of fibrous thickening with a variable content of VSMCs (Additional file 1: Figure S1).

**Fig. 1:**
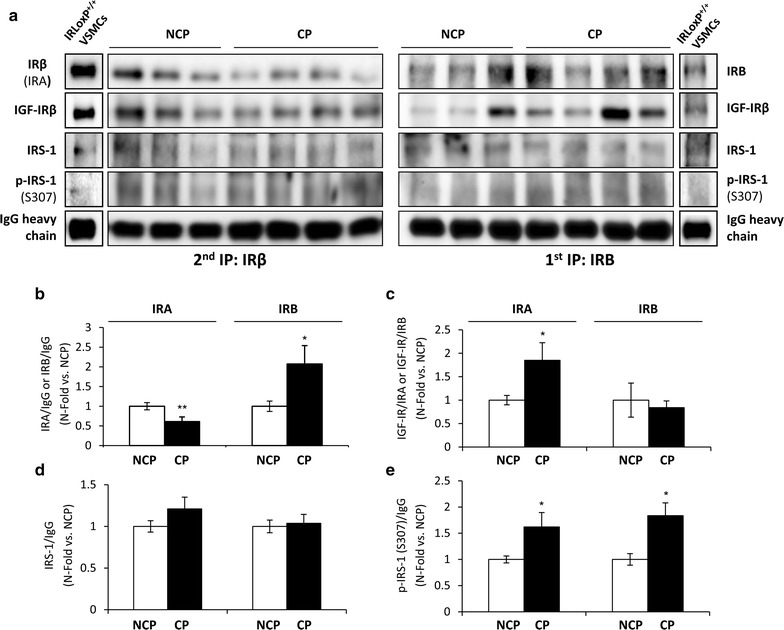
IR isoforms expression and association with IGF-IR or IRS-1 in human carotid atherosclerotic plaques. Representative gels (**a**) and quantifications (**b**–**e**) of IRA (left panels) and IRB (right panels) protein levels and their association with IGF-IR, IRS-1 or p-IRS-1 (Ser307) in non-complicated regions (n = 10) and their respective complicated plaques (n = 10). 150 µg of protein from each human plaque were immunoprecipitated with IRB isoform antibody. Supernatants were subsequently immunoprecipitated with IRβ (IRA and IRB isoforms) antibody. Immune complexes from first (only IRB) or second (only IRA) immunoprecipitations were collected on protein A-agarose beads and submitted to SDS-PAGE. IRLoxP^+/+^ VSMCs expressing both IR isoforms were used as an immunoprecipitation control. *p < 0.05, **p < 0.005 vs. NCP. *CP* complicated region of atherosclerotic plaque, *IP* immunoprecipitation, *NCP* non-complicated region

Firstly, the expression of each IR isoform and its association with IGF-IR or IRS-1 were comparatively analyzed in complicated and non-complicated regions of human plaques by consecutive immunoprecipitations of IRB and IRA isoforms (Fig. 1). We observed a significant decrease of IRA expression, together with an increase of IRB, in complicated regions (Fig. 1b). We also found that the expression of IRA/IGF-IR hybrid receptors was significantly higher in complicated regions of plaques (Fig. 1c). Regarding IRS-1 association with IRA or IRB, no significant differences were found in complicated as compared to non-complicated regions of the plaques (Fig. 1d).

**Fig. 2:**
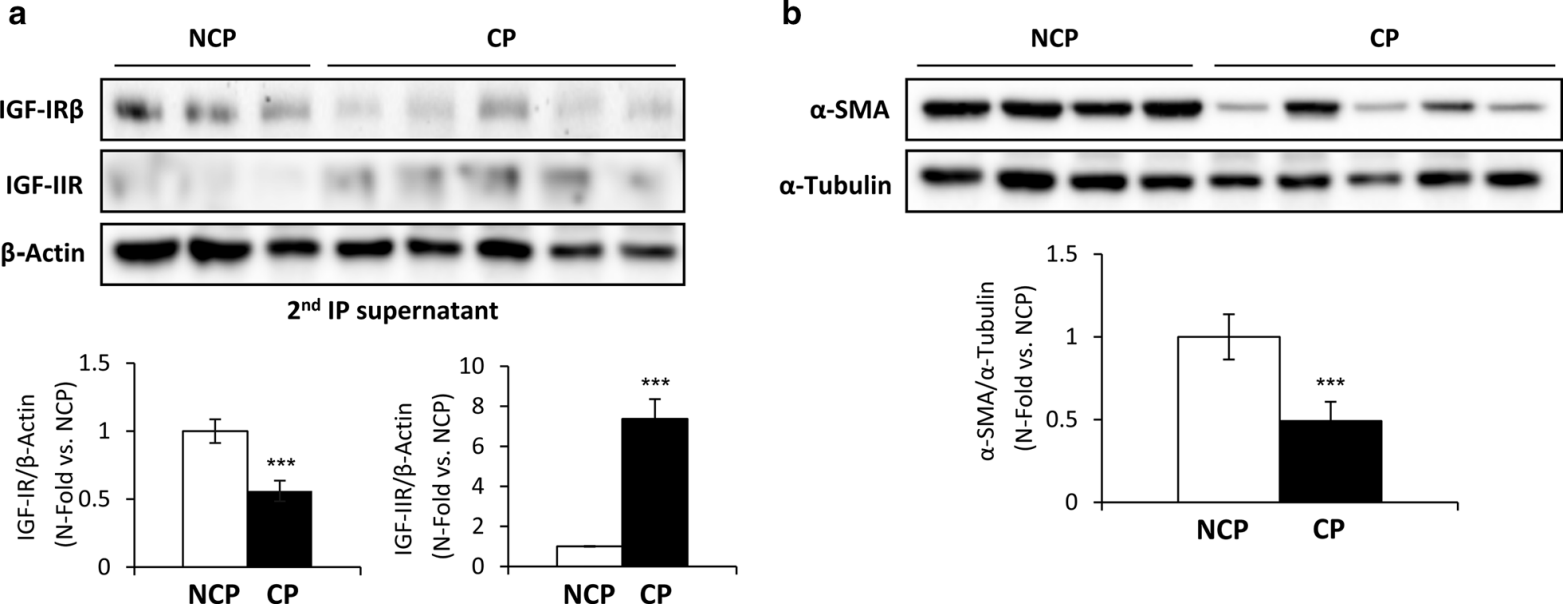
IGF-IR and IGF-IIR expression, and VSMCs content in human carotid atherosclerotic plaques. **a** Representative gels and quantifications of IGF-IR and IGF-IIR protein levels in supernatants from serial immunoprecipitations (IRB and IRA) of non-complicated regions (n = 10) and their respective complicated plaques (n = 10). **b** Analysis of VSMCs content by Western blot against α-SMA in non-complicated regions (n = 10) and their respective complicated plaques (n = 10). ***p < 0.0005 vs. NCP. *CP* complicated region of atherosclerotic plaque, *IP* immunoprecipitation, *NCP* non-complicated region

Nevertheless, IRA or IRB-associated IRS-1 showed an increased inhibitory Ser307 phosphorylation in complicated regions compared to non-complicated regions of plaques (Fig. 1e). Moreover, we observed a decreased insulin signaling in terms of Akt and p42/44 MAPK phosphorylation in complicated regions vs. non-complicated regions of atherosclerotic plaques (Additional file 2: Figure S2). To evaluate the expression of IGF-IR, we used the supernatants of the second immunoprecipitation in which this receptor would be as homodimers, since IR-associated IGF-IR was previously immunoprecipitated (Fig. 2a). We observed a reduced IGF-IR expression in complicated regions of the plaques. Conversely, the expression of IGF-IIR was significantly increased in complicated as compared to non-complicated regions of plaques (Fig. 2a).

In advanced atherosclerotic plaques, a loss of VSMCs as a result of their increased apoptosis may be key in plaque instability that seriously increases the risk of rupture. We therefore analyzed the content of VSMCs in human atherosclerotic plaques. Western blot analysis of α-smooth muscle actin (α-SMA), a well-known marker of smooth muscle cells, showed a significant reduction of VSMCs number in complicated as compared to non-complicated regions of plaques (Fig. 2b).

### Decrease of IRA/IRB ratio and less VSMCs content in experimental atherosclerotic models

**Fig. 3:**
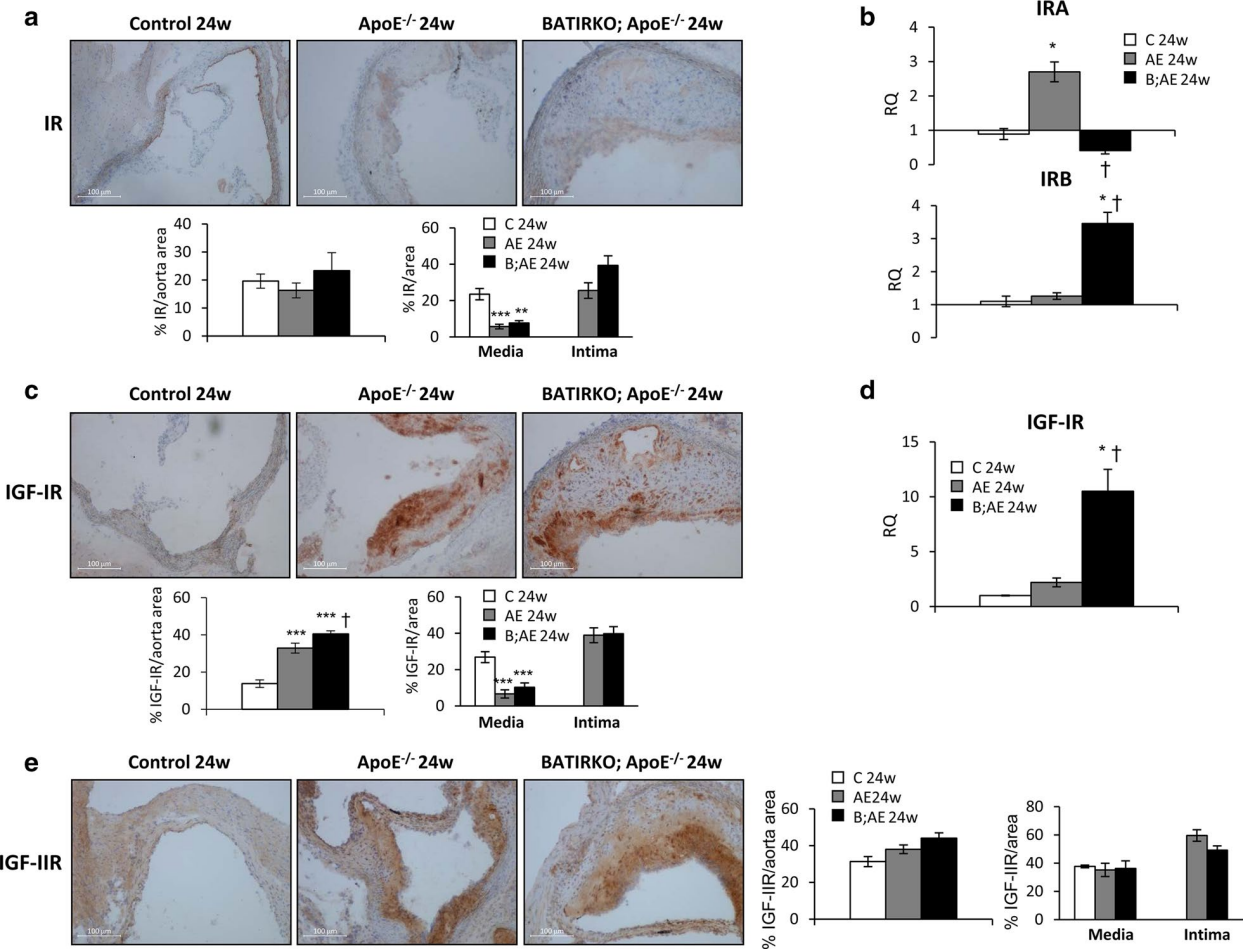
IR, IGF-IR and IGF-IIR expression in aorta from the 24-week-old model of experimental atherosclerosis. Representative photomicrographs and quantifications of immunohistochemistry against IR (**a**), IGF-IR (**c**) or IGF-IIR (**e**) in aortic roots from 24-week-old Control, ApoE^−/−^ and BATIRKO; ApoE^−/−^ mice. qRT-PCR analysis of IRA and IRB isoforms (**b**) or IGF-IR (**d**) expression in aorta from 24-week-old Control, ApoE^−/−^ and BATIRKO; ApoE^−/−^ mice. *p < 0.05, **p < 0.005, ***p < 0.0005 vs. Control mice; ^†^p < 0.05 vs. ApoE^−/−^ mice. C 24 weeks (n = 7); AE 24 weeks (n = 8); B;AE 24 weeks (n = 7). *AE* ApoE^−/−^, *B;AE* BATIRKO; ApoE^−/−^, *C* control

We wondered whether the findings obtained from human atherosclerotic plaques could also occur in experimental models of atherosclerosis. For this purpose, 24-week-old BATIRKO; ApoE^−/−^ mice under a Western type diet were used to develop more advanced atherosclerotic lesions as compared to Control and ApoE^−/−^ mice. These mice showed higher vascular damage, characterized by a significant increase of stenosis, lesion area, lipid content and macrophage infiltration in aortic roots, than 24-week-old ApoE^−/−^ mice [21]. In a second approach, Control, ApoE^−/−^ and BATIRKO; ApoE^−/−^ mice were fed on a standard diet and sacrificed at 15 months of age. Protein levels of IR in total area of aortic roots were very similar among the three groups of animals from the two experimental models (Fig. 3a and Additional file 3: Figure S3A). However, the receptor was significantly diminished in the media of aortic roots from ApoE^−/−^ and BATIRKO; ApoE^−/−^ mice in both 24-week-old and 15-month-old models. We observed by qRT-PCR that mRNA expression of IRA isoform was significantly decreased, whereas IRB was increased in aorta from 24-week-old BATIRKO; ApoE^−/−^ mice (Fig. 3b). These data are consistent with the reduced IRA/IRB ratio found in complicated human plaques (Fig. 1b). A significant increase of IGF-IR protein was observed in aortic roots from both 24-week-old and 15 month-old BATIRKO; ApoE^−/−^ mice in relation to their respective ApoE^−/−^ groups (Fig. 3c and Additional file 3: Figure S3B). Consistently, IGF-IR mRNA expression was significantly higher in aorta from 24-week-old BATIRKO; ApoE^−/−^ mice (Fig. 3d). IGF-IIR expression showed an increasing trend, although not significant, in aortic roots from 24-week-old mice BATIRKO; ApoE^−/−^ mice (Fig. 3e).

**Fig. 4:**
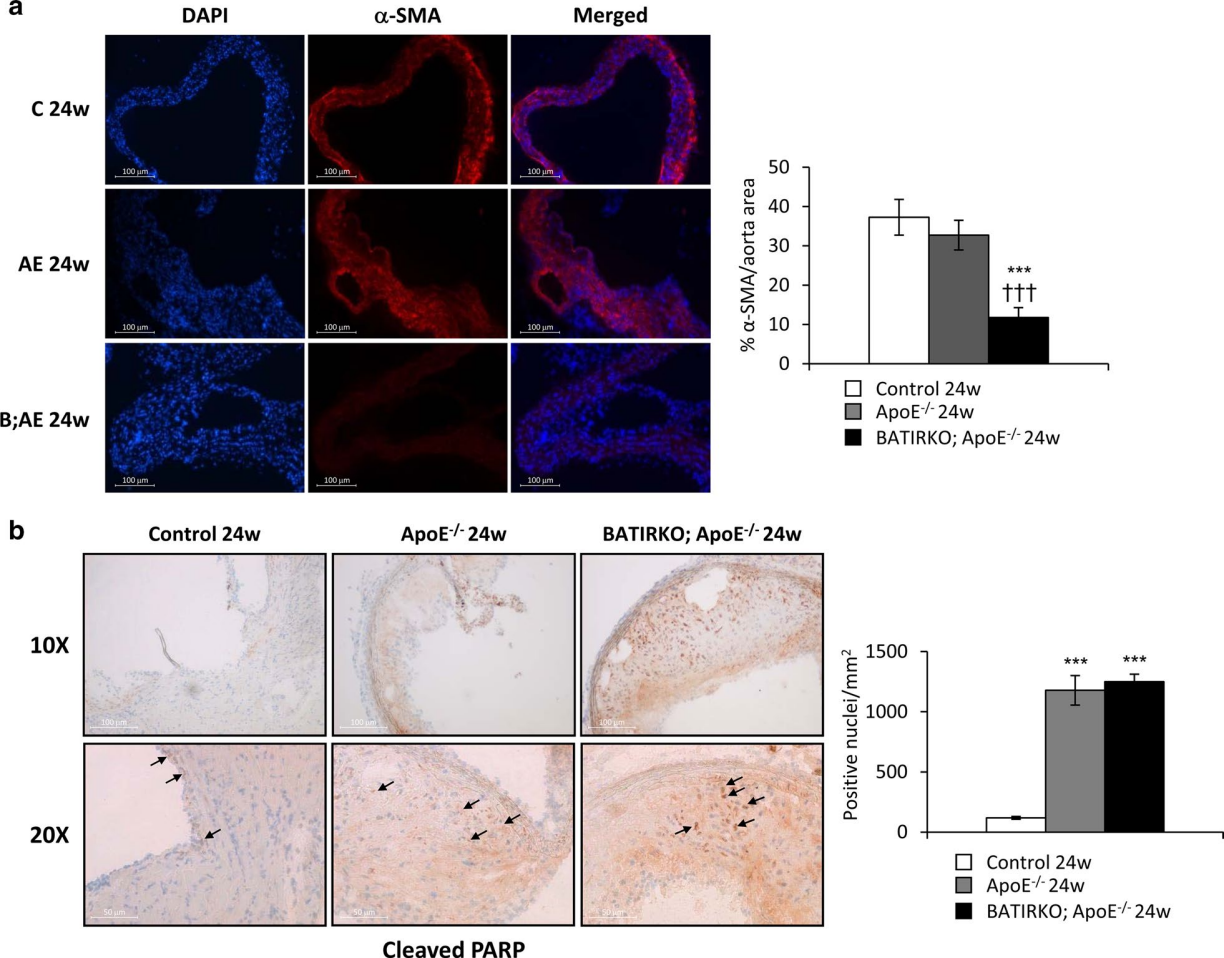
VSMCs content and apoptosis in atherosclerotic plaques from the 24-week-old experimental model. Representative photomicrographs and quantification of immunofluorescence against α-SMA (**a**) and of immunohistochemistry against cleaved PARP (**b**) in aortic roots from 24-week-old Control, ApoE^−/−^ and BATIRKO; ApoE^−/−^ mice. DAPI staining was performed to localize nuclei of cells presented in aortic roots (blue staining). ***p < 0.0005 vs. Control mice; ^†††^p < 0.0005 vs. ApoE^−/−^ mice. C 24 weeks (n = 7); AE 24 weeks (n = 8); B;AE 24 weeks (n = 7). *AE* ApoE^−/−^, *B;AE* BATIRKO; ApoE^−/−^, *C* control, *DAPI* 4′,6-diamidino-2-phenylindole, *PARP* poly ADP ribose polymerase, *α-SMA* α-smooth muscle actin

To study the VSMCs content in the atherosclerotic lesions of experimental models, we analyzed by immunohistochemistry the α-SMA expression in aortic roots from the different groups of animals. A very significant reduction of α-SMA was found in 24-week-old BATIRKO; ApoE^−/−^ mice as compared to the respective Control and ApoE^−/−^ groups (Fig. 4a). Moreover, atherosclerotic plaques from 15 month-old BATIRKO; ApoE^−/−^ mice also contained a significantly lower number of VSMCs than 15 month-old Control mice (Additional file 4: Figure S4A). An increased apoptosis is the major cause for the loss of VSMCs during atherogenesis [17]. We therefore evaluated the presence of apoptotic cells in aortic roots by immunostaining of the apoptosis marker cleaved poly ADP ribose polymerase (PARP). The amount of cleaved PARP^+^ cells was markedly increased in aortic roots from both 24-week-old ApoE^−/−^ and BATIRKO;ApoE^−/−^ mice in relation to 24-week-old Control mice (Fig. 4b). Similarly, aortic roots from 15 month-old ApoE^−/−^ and BATIRKO; ApoE^−/−^ mice had higher number of apoptotic cells than 15 month-old Control mice. In addition, the presence of apoptotic cells was significantly higher in 15 month-old BATIRKO; ApoE^−/−^ mice as compared to ApoE^−/−^ group (Additional file 4: Figure S4B).

### Antiapoptotic effect of IGF-IR on VSMCs

**Fig. 5:**
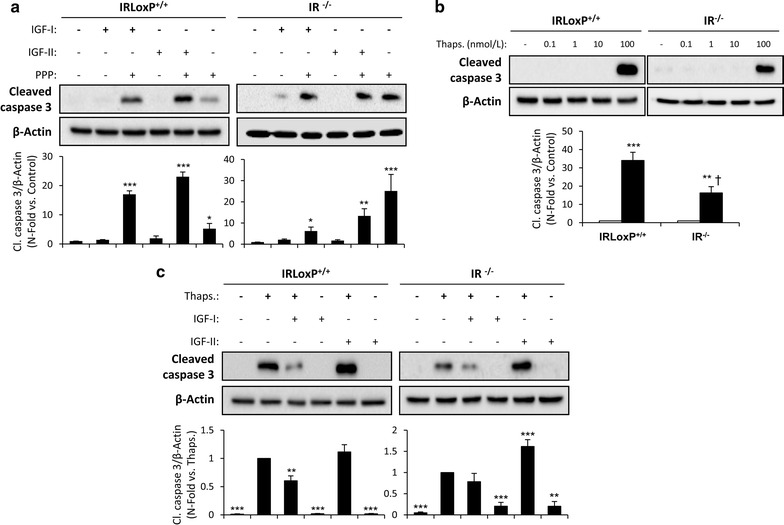
Antiapoptotic effect of IGF-IR on VSMCs. **a** Effect of IGF-IR inhibition by PPP (1 µmol/L) on cleaved caspase 3 levels analyzed by Western blot in IRLoxP^+/+^ and IR^−/−^ VSMCs stimulated with IGF-I (10 nmol/L) or IGF-II (10 nmol/L) for 24 h. **b** Dose–response effect of thapsigargin (0-100 nmol/L, 18 h) on cleaved caspase 3 levels in IRLoxP^+/+^ and IR^−/−^ VSMCs. **c** Effect of IGF-I or IGF-I pretreatment (10 nmol/L, 1 h) in IRLoxP^+/+^ and IR^−/−^ VSMCs treated with thapsigargin (100 nmol/L) for 18 h. Experiments were performed 4 or 5 times. *p < 0.05, **p < 0.005, ***p < 0.0005 vs. each control; ^†^p < 0.05 vs. IRLoxP^+/+^ VSMCs. *PPP* picropodophyllin

To assess the role of IR isoforms and IGF-IR in VSMCs apoptosis, four murine aortic VSMCs lines were used: bearing IR (IRLoxP^+/+^ VSMCs), lacking IR (IR^−/−^ VSMCs), expressing IRA isoform (IRA VSMCs) or alternatively expressing IRB isoform (IRB VSMCs). We previously demonstrated that IGF-IR is a main contributor to VSMC migration during early stages of atherosclerosis [14]. In the current work, we hypothesized that a decreased expression or activation of IGF-IR might promote apoptosis of VSMCs. To assure this, IRLoxP^+/+^ and IR^−/−^ VSMCs were treated with the highly specific inhibitor of IGF-IR tyrosine phosphorylation picropodophyllin (PPP) for 24 h. Western blot analysis of cleaved caspase 3 revealed that IGF-IR inhibition induced apoptosis in both cell lines (Fig. 5a). We also observed a significant increase of cleaved caspase 3 when cells were stimulated with IGF-I or IGF-II in the presence of PPP, this effect being greater with IGF-II stimulation (Fig. 5a). Afterwards, a dose–response curve of thapsigargin, an inhibitor of the endoplasmic reticulum (ER) Ca^2+^-ATPase inducing ER stress [24], was performed in IRLoxP^+/+^ and IR^−/−^ VSMCs. The expression of cleaved caspase 3 was found at a 100 nmol/L dose of thapsigargin in the two cell lines studied and this effect was significantly lower in IR^−/−^ VSMCs (Fig. 5b). Additionally, pretreatment with IGF-I, but not with IGF-II, reduced thapsigargin-induced apoptosis in IRLoxP^+/+^ and IR^−/−^ VSMCs measured by Western blot of active caspase 3 (Fig. 5c). In this sense, we also checked that IGF-I increased the percentage of viable cells and prevented from early and late apoptosis in IRLoxP^+/+^ and IR^−/−^ VSMCs (Additional file 5: Figure S5). These results suggest that IGF-I through IGF-IR has an antiapoptotic effect on VSMCs that may favor plaque stability by preventing the loss of those cells in atherosclerotic plaques.

**Fig. 6:**
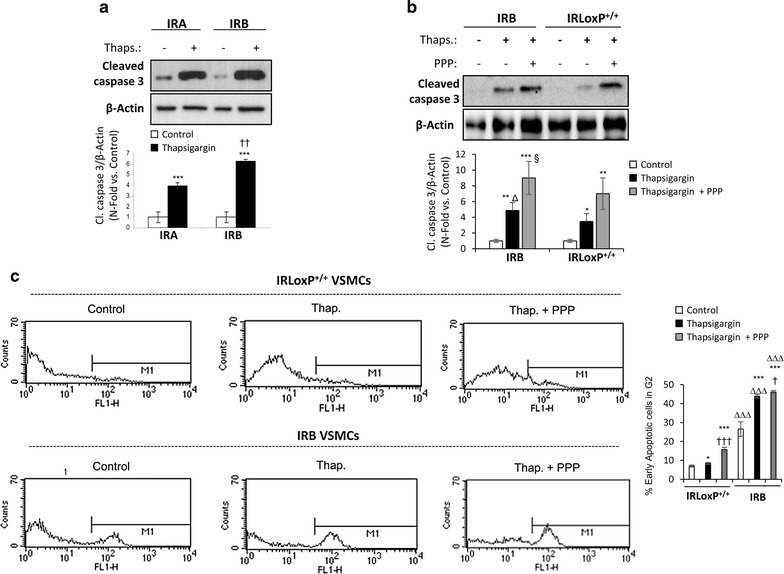
Differential contribution of IR isoforms to VSMCs apoptosis. **a** Effect of thapsigargin (100 nmol/L, 18 h) on cleaved caspase 3 levels analyzed by Western blot in IRA and IRB VSMCs. **b** Effect of IGF-IR inhibition by PPP (1 µmol/L) on cleaved caspase 3 levels in IRB and IRLoxP^+/+^ VSMCs treated with thapsigargin (100 nmol/L) for 18 h. Experiments were performed 4 times. *p < 0.05, **p < 0.005, ***p < 0.0005 vs. each control; ^††^p < 0.005 vs. IRA VSMCs; ^§^p < 0.05 vs. each thapsigargin; ^Δ^p < 0.05 vs. IRLoxP^+/+^ VSMCs. PPP: picropodophyllin. **c** % of early apoptotic cells in G2. These cells were stained positively for Annexin V-FITC and negatively for propidium iodide. *p < 0.05 vs. each control; ***p < 0.001 vs. each control; ^†^p < 0.05 vs. each thapsigargin; ^†††^p < 0.001 vs. each thapsigargin; ^ΔΔΔ^p < 0.001 vs. each point of IRLoxP^+/+^ VSMCs

**Fig. 7:**
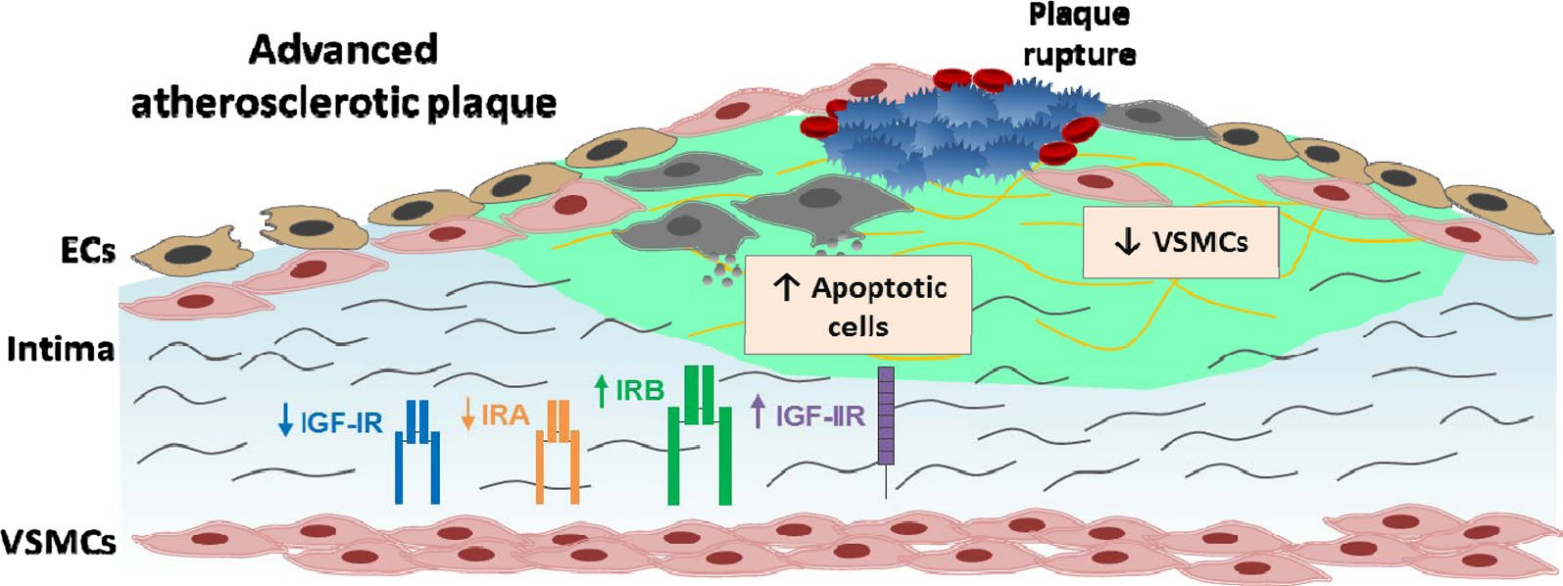
Proposed scheme of an advanced atherosclerotic plaque showing the expression profiles of IR isoforms (IRA and IRB), IGF-IR and IGF-IIR. We found a decreased IRA/IRB ratio, increased IGF-IIR expression, and reduced IGF-IR expression, as well as lower VSMCs content in complicated versus non-complicated regions of human carotid plaques. In experimental models developing advanced atherosclerotic plaques, we also observed a reduced IRA/IRB ratio, a decrease of VSMCs content and an increase of apoptotic cell number. Finally, our in vitro results show that a decrease of IGF-IR expression and the presence of IRB as the predominant IR isoform increase the apoptosis of VSMCs. Therefore, this scenario could contribute to a loss of VSMCs, promoting plaque instability and leading to a higher risk of plaque rupture with the outcome of vascular complications aggravation. *ECs* endothelial cells, *VSMCs* vascular smooth muscle cells

Based on the reduced IRA/IRB ratio observed in complicated human plaques and in advanced experimental atherosclerosis, we wondered whether IR isoforms could have a differential contribution to VSMCs apoptosis. For this purpose, we studied the apoptosis induced by thapsigargin in IRA and IRB VSMCs. Cells exclusively expressing IRB showed a significantly higher increase of cleaved caspase 3 as compared to IRA VSMCs (Fig. 6a). Finally, IGF-IR inhibition by PPP enhanced the thapsigargin-induced apoptosis of IRB VSMCs in a significant way (Fig. 6b, c and Additional file 6: Figure S6). However, in IRLoxP^+/+^ VSMCs, expressing both IR isoforms, PPP caused a lower increase of cleaved caspase 3 levels (Fig. 6b, c and Additional file 6: Figure S6). These data indicate that, in addition to IGF-IR inhibition, decreased IRA/IRB ratio may contribute to VSMCs apoptosis and thereby to plaque instability (Fig. 7).

## Discussion

In early stages of atherosclerosis, abnormal proliferation and migration of VSMCs is a major contributor to atherogenesis. By contrast, VSMCs in advanced lesions are generally regarded as having atheroprotective plaque-stabilizing properties [3]. Indeed, clinical studies have reported that atherosclerotic plaques with a relative decrease of VSMCs and a thin fibrous cap, as well as increased inflammatory cells, were less stable and more prone to rupture [25, 26]. In this sense, it has been described that VSMCs present in human atherosclerotic lesions showed higher apoptosis than those derived from normal vessels [27].

The IR isoforms possess different functional features. Whereas IRB upregulation is associated with predominant metabolic insulin signaling, an increased expression of IRA is associated with decreased metabolic insulin signaling and increased IGF signaling, favoring cell growth, proliferation and survival [8]. We previously described that overexpression of IRA isoform favors VSMCs proliferation and migration contributing to atherosclerotic progression [13, 14]. However, to our knowledge, no data are available regarding the expression profile of IR isoforms in advanced atherosclerotic lesions and their role in VSMCs apoptosis. In the current paper, we have shown a decreased IRA/IRB ratio in complicated regions of human atherosclerotic plaques. Since IRA isoform binds IGF-II with higher affinity than IRB [7], its overexpression may promote proatherogenic effects of IGF-II in early stages of atherosclerotic process. Conversely, in more advanced stages, in which a local action of IGF-II prevents plaque instability by promoting the proliferation of intimal VSMCs [28], a reduced IRA/IRB ratio could favor the destabilization of atherosclerotic plaques. Although there were no significant changes on IRS-1 association with IR isoforms, its serine phosphorylation was increased as well as Akt and p42/44 MAPK phosphorylation were diminished in complicated regions of human plaques. The phosphorylation of IRS-1 on serine residues is associated with insulin resistance induced by cytokines such as TNF-α, since it inhibits IRS-1 tyrosine phosphorylation leading to impaired insulin signaling [29]. In addition, the increase of IRA/IGF-IR hybrid receptors in complicated regions might also be contributing to vascular insulin resistance since it has been previously described IRs organized as holoreceptors favor insulin signaling [30]. There is ample clinical evidence that insulin resistance increases the risk for cardiovascular diseases even in the absence of hyperglycemia. Insulin resistance syndromes can promote both atherogenesis and advanced plaque progression, and the mechanisms likely involve both systemic factors, particularly dyslipidemia, hypertension, and a pro-inflammatory state, but also the effect of perturbed insulin signaling at the level of the intimal cells that participate in atherosclerosis, including endothelial cells, VSMCs, and macrophages [31]. In this sense, atherosclerotic plaques from diabetic subjects had larger necrotic cores, greater infiltration of inflammatory cells and increased apoptotic VSMCs and macrophages than nondiabetic subjects [32].

We also observed a decreased expression of IGF-IR in complicated as compared to non-complicated regions of human carotid plaques. IGF-I through IGF-IR prevents apoptosis, and promotes extracellular matrix formation, proliferation and migration of VSMCs. Furthermore, it has been described that IGF-I was less potent in inducing the survival of VSMCs from the endarterectomy specimens of symptomatic patients than those of asymptomatic subjects [33]. In this context, a decreased expression of IGF-I and IGF-IR in advanced atherosclerotic plaques has been involved in VSMCs apoptosis leading to plaque instability and rupture [18–20]. By contrast, IGF-IIR was increased in complicated human carotid plaques. There are evidences showing that in addition to lysosomal degradation of IGF-II [9], IGF-IIR may trigger intracellular signaling cascades involved in cell behavior regulation [10, 11]. Deficiency of IGF-IIR in transgenic mice leads to ventricular hyperplasia in heart with an increased proliferation of fetal cardiomyocytes [34]. On the other hand, an increased cardiac expression of IGF-II and IGF-IIR and cardiomyocyte apoptosis were found in hypertensive rats with abdominal aorta ligation [35]. Other studies have supported the idea that IGF-II, acting through IGF-IIR, may promote the death of cardiac myocytes [36–38]. According to that data, the upregulation of IGF-IIR in atherosclerotic lesions could contribute to plaque instability by two potential mechanisms: reduced IGF-II bioavailability and thereby less effect on cell survival, or increased apoptosis of plaque-resident cells. Several studies have demonstrated the presence of apoptosis in human atherosclerotic plaques as the leading cause of loss of VSMCs [27, 39, 40]. Thus, unstable plaques from coronary atherectomy contained less apoptotic VSMCs and a reduced total cell number than stable plaques [41]. Consistently, we have described a decrease of VSMCs content in complicated human atherosclerotic plaques in relation to non-complicated regions. However, one of the limitations of this study is the small number of patients included, since it is difficult to obtain samples of the complicated and non-complicated regions of atherosclerotic plaques from the same patient. Therefore, in order to validate our results from human atherosclerotic plaques, additional studies including a greater number of patients need to be performed.

To assess whether the results obtained from human atherosclerotic plaques could be found in experimental atherosclerotic lesions, we used 24-week-old and 15-month-old BATIRKO; ApoE^−/−^ mice that showed more advanced atherosclerotic plaques than ApoE^−/−^ mice. Nevertheless, although hyperlipidemic genetically manipulated models such as ApoE^−/−^ mice developed the entire spectrum of lesions similar to those of humans, their major limitation is the infrequency of plaque rupture and thrombosis, two common complications of human atherosclerosis [42]. Therefore, some, but not all, of the findings from complicated regions of human plaques were also observed in atherosclerosis plaques from BATIRKO; ApoE^−/−^ mice: decreased IRA/IRB ratio, increasing trend of IGF-IIR and reduced VSMC content with an increase of apoptotic cells. However, IGF-IR expression was increased in total area of aortic roots, but decreased in the media, in both ApoE^−/−^ and BATIRKO; ApoE^−/−^ mice vs. Control mice.

Our in vitro results indicate an antiapoptotic role of IGF-IR in VSMCs. The inhibition of IGF-IR by PPP induced apoptosis in IRLoxP^+/+^ and IR^−/−^ VSMCs. We previously observed that the lack of IR in VSMCs led to an increased expression of IGF-IR [14], and this seems to be responsible for the lower thapsigargin-induced apoptosis that we found in IR^−/−^ VSMCs as compared to IRLoxP^+/+^ VSMCs. Moreover, IGF-I, but not IGF-II, exerts a protective effect on the thapsigargin-induced apoptosis of VSMCs. Consistently, VSMCs from human atherosclerotic plaques showed an intrinsic sensitivity to apoptosis caused in part by defective expression of IGF-IR, impaired IGF-I-mediated survival signaling and increased IGFBP secretion [19]. Oxidized LDL uptake has been proposed as a mechanism triggering apoptosis in VSMCs mediated by downregulation of IGF-IR [43]. Thus, overexpression of IGF-IR by using an adenovirus completely abrogated this effect on human VSMCs [44]. By contrast, IGF-II not only does not prevent VSMCs apoptosis, but also appears to enhance thapsigargin-induced apoptosis in VSMCs lacking IR. This effect could be explained by IGF-II binding to IGF-IIR that, as mentioned above, has been reported to promote apoptosis in cardiomyocytes [33–36]. Finally, we found a differential contribution of IR isoforms to VSMCs apoptosis, since cells exclusively expressing IRB were more prone to thapsigargin-induced apoptosis than IRA or IRLoxP^+/+^ VSMCs.

Overall, the data in this study advance our understanding of the pathophysiology of human carotid atherothrombosis and the potential role of IR isoforms and IGFs receptors in the stability of atherosclerotic plaques. Thus, our findings suggest that manipulation of the IGF-I autocrine/paracrine pathway may be a useful strategy to limit the loss of VSMCs that contribute to atherosclerotic plaque destabilization.

## Conclusions

In conclusion, our results show for the first time a decreased IRA/IRB ratio and increased IGF-IIR expression as well as reduced IGF-IR expression in complicated versus non-complicated regions of human atherosclerotic plaques (Fig. 7). These events may contribute to an increased apoptosis and subsequent loss of VSMCs, promoting plaque instability and leading to a higher risk of plaque rupture with the outcome of vascular complications aggravation.

## Additional files

**Additional file 1: Figure S1.** Histological analysis of human carotid atherosclerotic plaques. Masson’s trichrome stain of representative non-complicated and complicated plaques of human carotid atherosclerosis. Different regions of the plaque are shown: M: media; F: fibrous region; A: atheroma; S: shoulder.

**Additional file 2: Figure S2.** Differential insulin signaling in complicated and non-complicated regions from atherosclerotic plaques. Western blot analysis of phosphorylation of Akt (**A**) and p42/44 MAPK (**B**) protein levels in supernatants from serial immunoprecipitations (IRB and IRA) of non-complicated regions (n = 10) and their respective complicated regions (n = 10). CP: complicated region of atherosclerotic plaque; IP: immunoprecipitation; NCP: non-complicated region. *p < 0.05 vs. NCP; **p < 0.001 vs. NCP.

**Additional file 3: Figure S3.** IR and IGF-IR expression in aorta from the 15-month-old model of experimental atherosclerosis. Representative photomicrographs and quantifications of immunohistochemistry against IR (**A**) or IGF-IR (**B**) in aortic roots from 15-month-old Control, ApoE^−/−^ and BATIRKO; ApoE^−/−^ mice. **p < 0.005, ***p < 0.0005 vs. Control mice; ^††^p < 0.005 vs. ApoE^−/−^ mice. C 15 m (n = 7); AE 15 m (n = 6); B; AE 15 m (n = 6). AE: ApoE^−/−^, B;AE: BATIRKO; ApoE^−/−^; C: Control.

**Additional file 4: Figure S4.** VSMC content and apoptosis in atherosclerotic plaques from the 15-month-old experimental model. Representative photomicrographs and quantification of immunofluorescence against α-SMA (**A**) and of immunohistochemistry against cleaved PARP (**B**) in aortic roots from 15-month-old Control, ApoE^−/−^ and BATIRKO; ApoE^−/−^ mice. DAPI staining was performed to localize nuclei of cells presented in aortic roots (blue staining). *p < 0.05, ***p < 0.0005 vs. Control mice; ^†^p < 0.05 vs. ApoE^−/−^ mice. C 15 m (n = 7); AE 15 m (n = 6); B;AE 15 m (n = 6). AE: ApoE^−/−^, B;AE: BATIRKO; ApoE^−/−^; C: Control; DAPI: 4′,6-diamidino-2-phenylindole; PARP: poly ADP ribose polymerase; α-SMA: α-smooth muscle actin.

**Additional file 5: Figure S5.** Antiapoptotic effect of IGF-IR and IGF-I on IRLoxP^+/+^ and IR^−/−^ VSMCs. Analysis of dead cells by Annexin V-FITC and propidium iodide assays. (**A**) Representative images of % necrotic cells (UL), % dead cells (UR), % viable cells (LL) and % early apoptotic cells (LR) in G1 Gate. FL1H (x-axis, Annexin V FITC); FL3H (y-axis, propidium iodide). Graphics of % of viable, % of apoptotic cells (**B**), % of necrotic cells, % of dead cells and % of early apoptotic cells (**C**). *p < 0.05 vs. each Control; **p < 0.001 vs. each Control; **p < 0.0001 vs. each control; ^†^p < 0.05 vs. each thapsigargin; ^†††^p < 0.0001 vs. each thapsigargin.

**Additional file 6: Figure S6.** Differential apoptotic effects of thapsigargin and IGF-IR inhibitor on IRLoxP^+/+^ and IRB VSMCs. Analysis of dead cells by Annexin V-FITC and propidium iodide assays. (**A**) Representative images of % necrotic cells (UL), % dead cells (UR), % viable cells (LL) and % early apoptotic cells (LR) in G1 Gate. FL1H (x-axis, Annexin V FITC); FL3H (y-axis, propidium iodide). Graphics of % of viable, % of apoptotic cells (**B**), % of necrotic cells, % of dead cells and % of early apoptotic cells (**C**). *p < 0.05 vs. each control; **p < 0.001 vs. each control; **p < 0.0001 vs. each control; ^†^p < 0.05 vs. each thapsigargin; ^†††^p < 0.0001 vs. each thapsigargin.

## Abbreviations

α-SMA: α-smooth muscle actin
ApoE^−/−^: apolipoprotein E knockout
BATIRKO: brown adipose tissue-specific insulin receptor knockout
CP: complicated plaque
DAPI: 4′,6-diamidino-2-phenylindole
IGF-I: insulin-like growth factor-I
IGF-II: insulin-like growth factor-II
IGF-IIR: insulin-like growth factor-II receptor
IGF-IR: insulin-like growth factor-I receptor
IGFs: insulin-like growth factors
IR: insulin receptor
IRA: insulin receptor A isoform
IRB: insulin receptor B isoform
IRS-1: insulin receptor substrate 1
NCP: non-complicated region of the plaque
PARP: poly ADP ribose polymerase
PPP: picropodophyllin
qRT-PCR: real-time quantitative PCR
VSMCs: vascular smooth muscle cells
