# Supplementary figures and images for: Potential role of insulin receptor isoforms and IGF receptors in plaque instability of human and experimental atherosclerosis
Source: Cardiovasc Diabetol. 2018 Feb 20;17:31. doi: 10.1186/s12933-018-0675-2 (PMC5819698; doi:10.1186/s12933-018-0675-2)

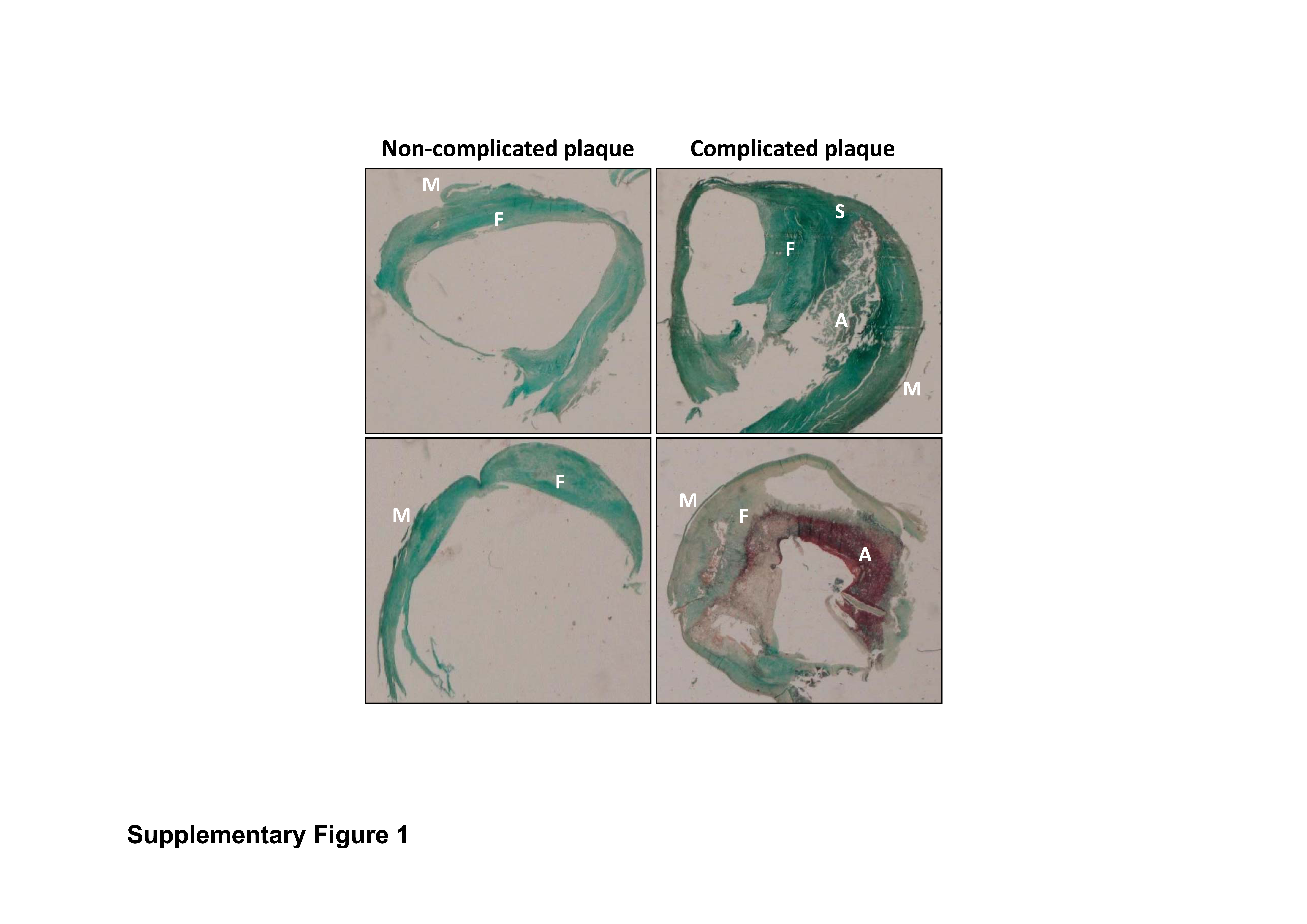

Supplement: Supplementary file 1 — Additional file 1: Figure S1. Histological analysis of human carotid atherosclerotic plaques. Masson’s trichrome stain of representative non-complicated and complicated plaques of human carotid atherosclerosis. Different regions of the plaque are shown: M: media; F: fibrous region; A: atheroma; S: shoulder. [file 12933_2018_675_MOESM1_ESM.tif]

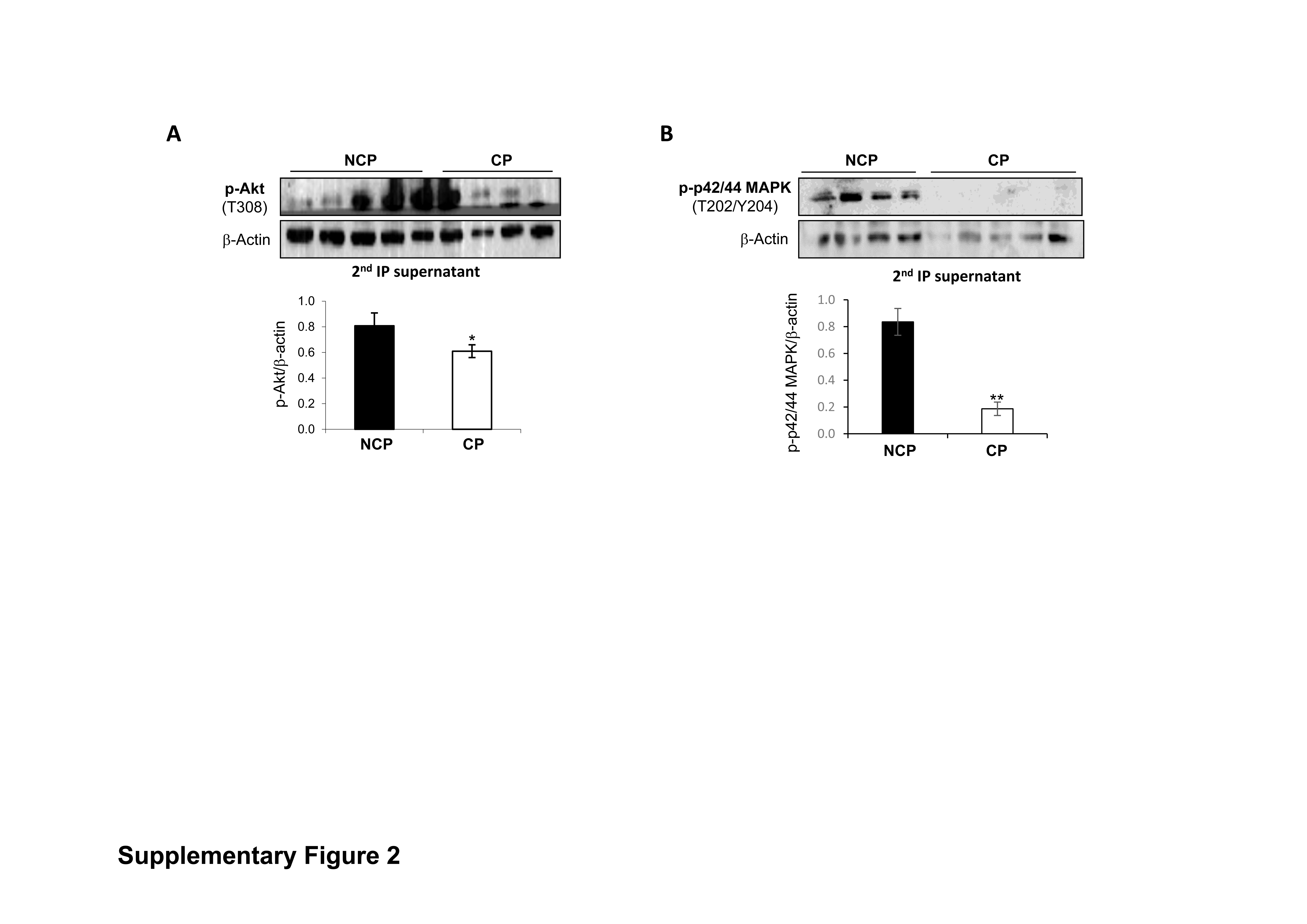

Supplement: Supplementary file 2 — Additional file 2: Figure S2. Differential insulin signaling in complicated and non-complicated regions from atherosclerotic plaques. Western blot analysis of phosphorylation of Akt (A) and p42/44 MAPK (B) protein levels in supernatants from serial immunoprecipitations (IRB and IRA) of non-complicated regions (n = 10) and their respective complicated regions (n = 10). CP: complicated region of atherosclerotic plaque; IP: immunoprecipitation; NCP: non-complicated region. *p < 0.05 vs. NCP; **p < 0.001 vs. NCP. [file 12933_2018_675_MOESM2_ESM.tif]

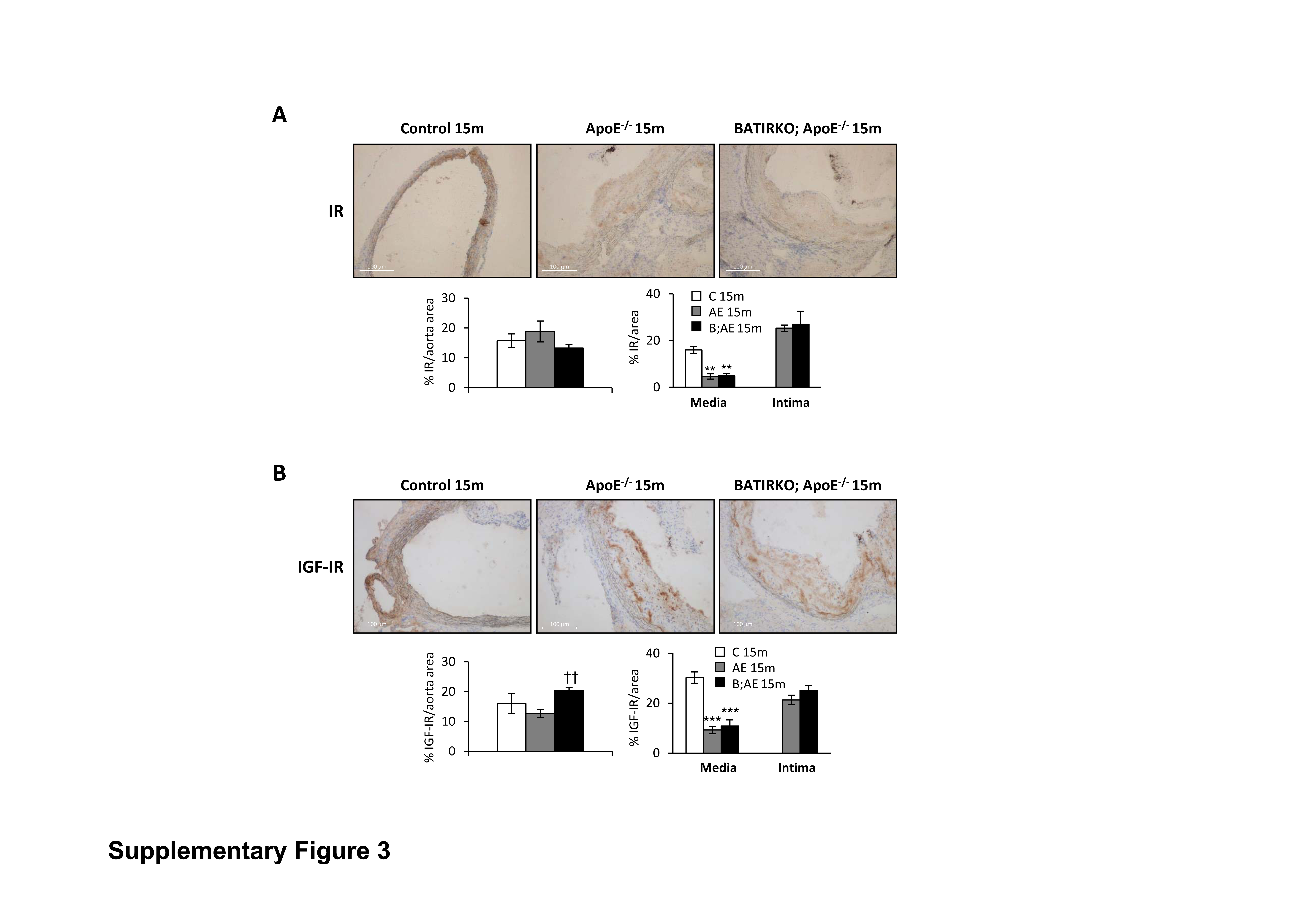

Supplement: Supplementary file 3 — Additional file 3: Figure S3. IR and IGF-IR expression in aorta from the 15-month-old model of experimental atherosclerosis. Representative photomicrographs and quantifications of immunohistochemistry against IR (A) or IGF-IR (B) in aortic roots from 15-month-old Control, ApoE−/− and BATIRKO; ApoE−/− mice. **p < 0.005, ***p < 0.0005 vs. Control mice; ††p < 0.005 vs. ApoE−/− mice. C 15 m (n = 7); AE 15 m (n = 6); B; AE 15 m (n = 6). AE: ApoE−/−, B;AE: BATIRKO; ApoE−/−; C: Control. [file 12933_2018_675_MOESM3_ESM.tif]

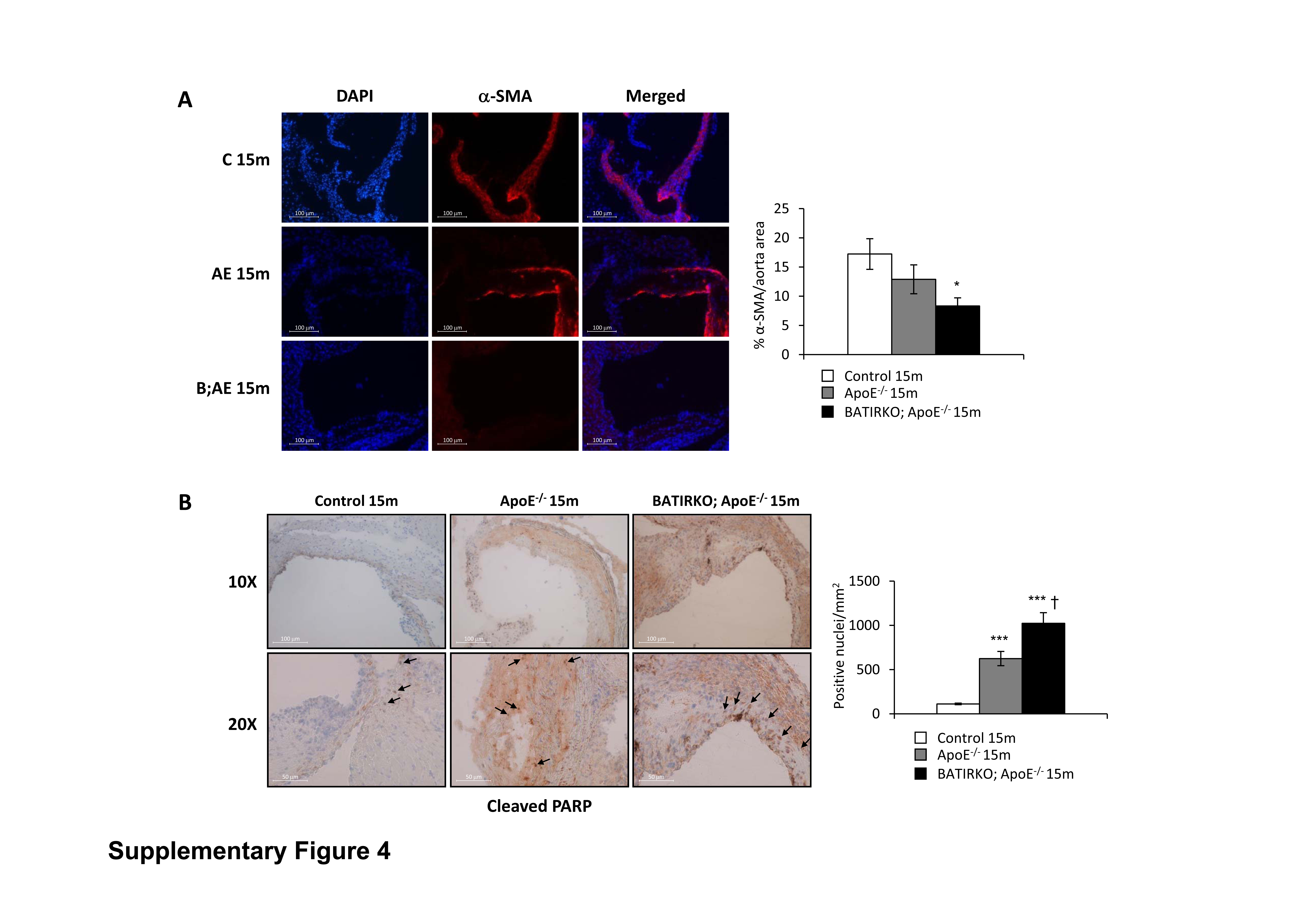

Supplement: Supplementary file 4 — Additional file 4: Figure S4. VSMC content and apoptosis in atherosclerotic plaques from the 15-month-old experimental model. Representative photomicrographs and quantification of immunofluorescence against α-SMA (A) and of immunohistochemistry against cleaved PARP (B) in aortic roots from 15-month-old Control, ApoE−/− and BATIRKO; ApoE−/− mice. DAPI staining was performed to localize nuclei of cells presented in aortic roots (blue staining). *p < 0.05, ***p < 0.0005 vs. Control mice; †p < 0.05 vs. ApoE−/− mice. C 15 m (n = 7); AE 15 m (n = 6); B;AE 15 m (n = 6). AE: ApoE−/−, B;AE: BATIRKO; ApoE−/−; C: Control; DAPI: 4′,6-diamidino-2-phenylindole; PARP: poly ADP ribose polymerase; α-SMA: α-smooth muscle actin. [file 12933_2018_675_MOESM4_ESM.tif]

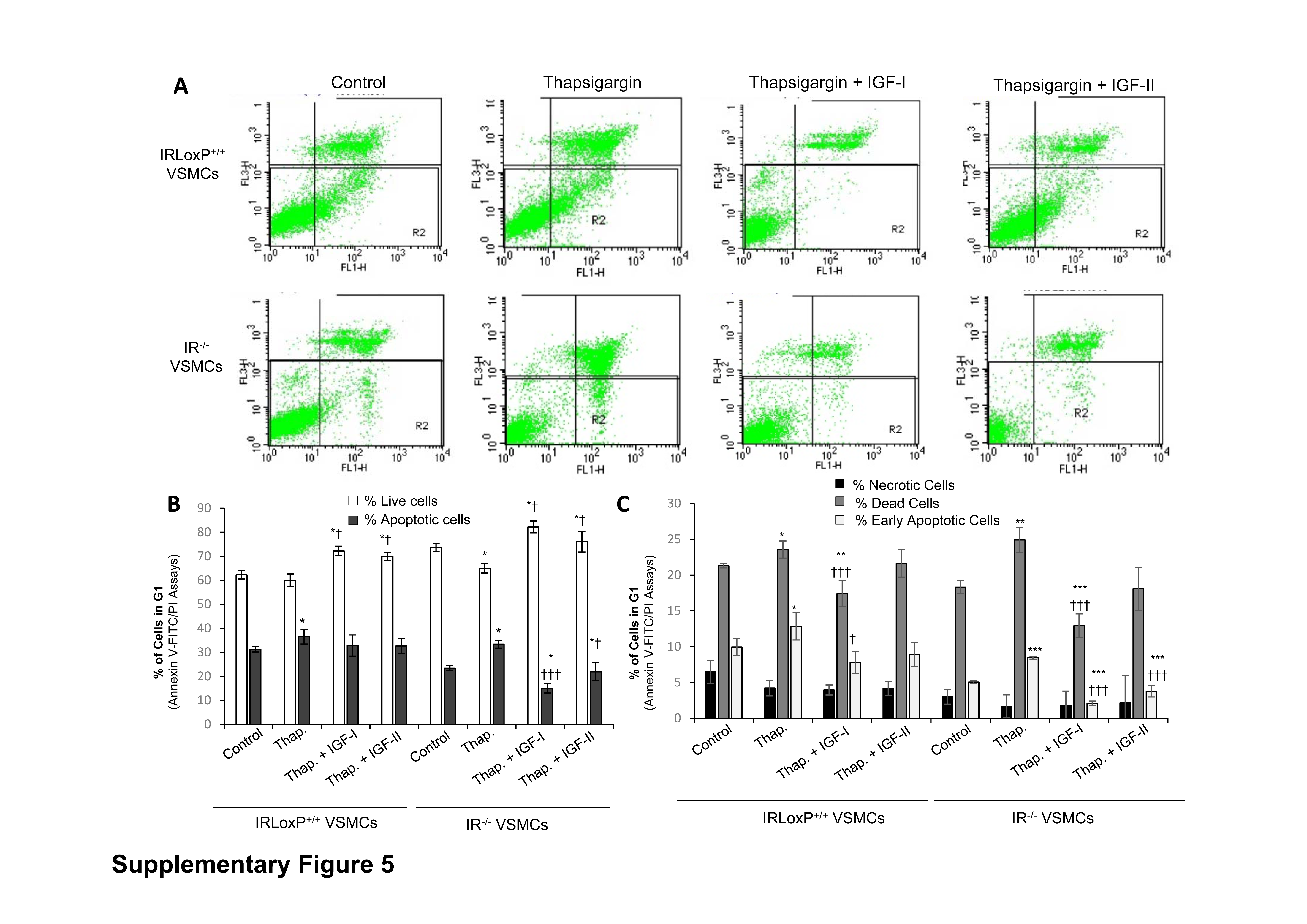

Supplement: Supplementary file 5 — Additional file 5: Figure S5. Antiapoptotic effect of IGF-IR and IGF-I on IRLoxP+/+ and IR−/− VSMCs. Analysis of dead cells by Annexin V-FITC and propidium iodide assays. (A) Representative images of % necrotic cells (UL), % dead cells (UR), % viable cells (LL) and % early apoptotic cells (LR) in G1 Gate. FL1H (x-axis, Annexin V FITC); FL3H (y-axis, propidium iodide). Graphics of % of viable, % of apoptotic cells (B), % of necrotic cells, % of dead cells and % of early apoptotic cells (C). *p < 0.05 vs. each Control; **p < 0.001 vs. each Control; **p < 0.0001 vs. each control; †p < 0.05 vs. each thapsigargin; †††p < 0.0001 vs. each thapsigargin. [file 12933_2018_675_MOESM5_ESM.tif]

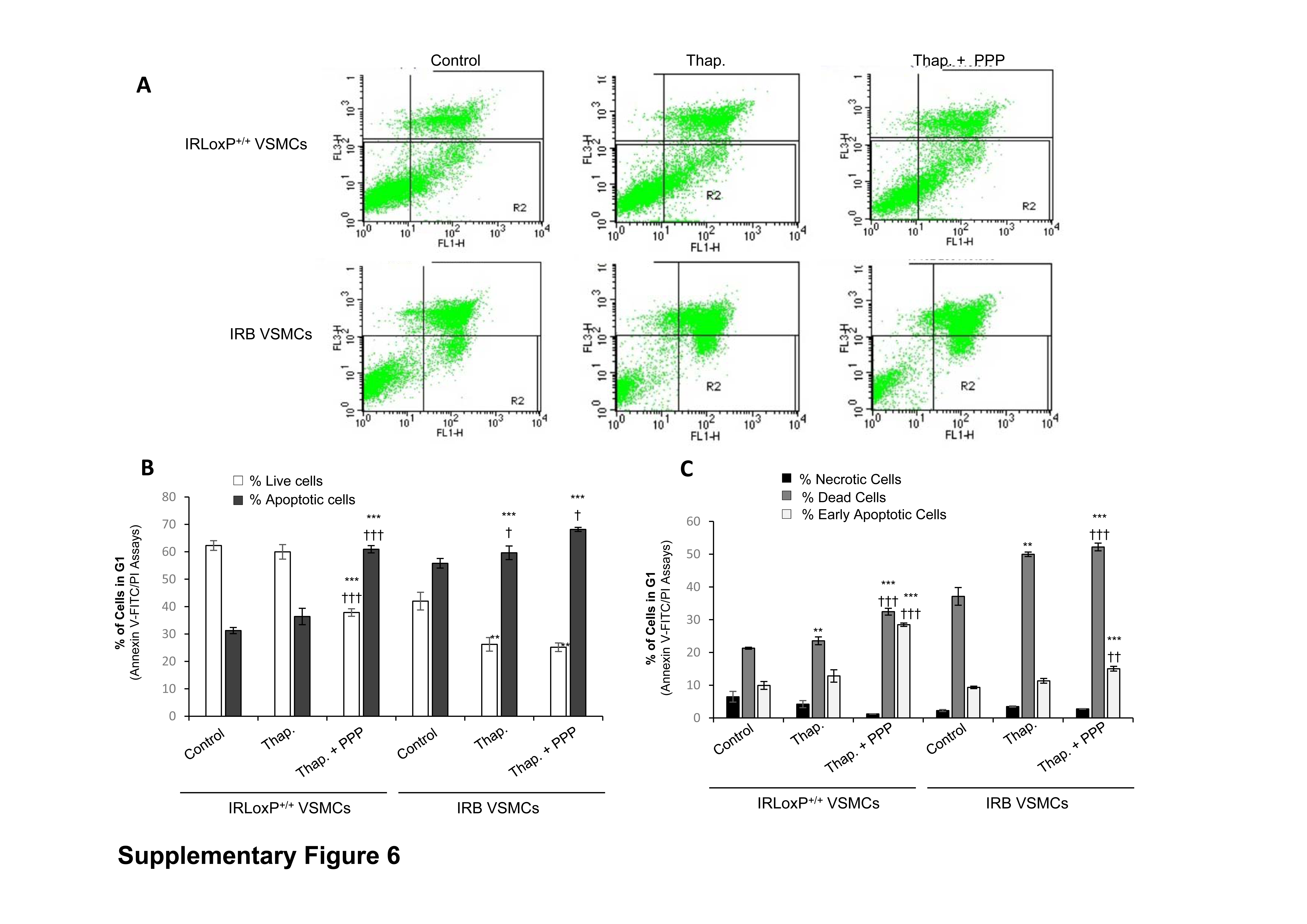

Supplement: Supplementary file 6 — Additional file 6: Figure S6. Differential apoptotic effects of thapsigargin and IGF-IR inhibitor on IRLoxP+/+ and IRB VSMCs. Analysis of dead cells by Annexin V-FITC and propidium iodide assays. (A) Representative images of % necrotic cells (UL), % dead cells (UR), % viable cells (LL) and % early apoptotic cells (LR) in G1 Gate. FL1H (x-axis, Annexin V FITC); FL3H (y-axis, propidium iodide). Graphics of % of viable, % of apoptotic cells (B), % of necrotic cells, % of dead cells and % of early apoptotic cells (C). *p < 0.05 vs. each control; **p < 0.001 vs. each control; **p < 0.0001 vs. each control; †p < 0.05 vs. each thapsigargin; †††p < 0.0001 vs. each thapsigargin. [file 12933_2018_675_MOESM6_ESM.tif]
